# The impact of sarcopenia on survival and treatment tolerance in patients with head and neck cancer treated with chemoradiotherapy

**DOI:** 10.1002/cam4.5278

**Published:** 2022-10-20

**Authors:** Rita Bentahila, Philippe Giraud, Pierre Decazes, Sarah Kreps, Paula Nay, Augustin Chatain, Emmanuelle Fabiano, Catherine Durdux

**Affiliations:** ^1^ Department of Radiation Oncology European Georges Pompidou Hospital Paris France; ^2^ Department of Nuclear Medicine Henri Becquerel Cancer Center Rouen France

**Keywords:** head and neck cancer, radiotherapy, sarcopenia, survival, toxicities

## Abstract

**Background:**

Sarcopenia appears to be a negative prognostic factor for poor survival outcomes and worse treatment tolerance in patients with head‐and‐neck squamous cell carcinoma (HNSCC). We evaluated sarcopenia's impact on overall survival (OS), disease‐free survival (DFS) and chemo‐radiation tolerance in patients with head‐and‐neck cancer (HNC) treated with chemoradiotherapy (CRT) from a monocentric observational study.

**Methods:**

We identified patients with HNC treated by CRT between 2009 and 2018 with pretreatment imaging using positron emission tomography–computed tomography scans (PET/CT). Sarcopenia was measured using the pretreatment PET/CT at the L3 vertebral body using previously published methods. Clinical variables were retrospectively retrieved.

**Results:**

Of 216 patients identified, 54 patients (25.47%) met the criteria for sarcopenia. These patients had a lower mean body mass index before treatment (21.92 vs. 25.65 cm/m^2^, *p* < 0.001) and were more likely to have a history of smoking (88.89% vs. 71.52%, *p* = 0.01), alcohol use (55.56% vs. 38.61%, *p* = 0.03) and positive human papilloma virus status (67.74% vs. 41.75%, *p* = 0.011). At 3 years of follow‐up, OS and DFS were 75% and 70% versus 82% and 85% for sarcopenic and non‐sarcopenic patients, respectively (*p* = 0.1 and *p* = 0.00015). On multivariate analysis, sarcopenia appeared as a pejorative factor on DFS (hazard ratio 2.174, *p* = 0.0001) in the overall cohort. Sarcopenic patients did not require more chemotherapy and radiation‐treatment interruptions and did not suffer from more chemo‐induced and radiation‐induced grade 3–4 toxicities than their non‐sarcopenic counterparts.

**Conclusion:**

Sarcopenia in HNSCC patients is an independent adverse prognostic factor for DFS after definitive chemoradiotherapy.

## Abstract 2

Sarcopenia in patients with head‐and‐neck squamous cell carcinoma treated by definitive chemoradiotherapy is an independent negative prognostic factor for disease‐free survival. Sarcopenic patients did not have more grade 3+ toxicities or more treatment interruptions.

## 1 INTRODUCTION

In recent years, sarcopenia has been reported as a predictive prognostic factor in malignancies.^1^, ^2^ Sarcopenia is characterized by progressive and generalized loss of skeletal muscle mass (SMM) and function.^3^ Measurement of the SMM using computed tomography (CT) imaging at the level of the third lumbar vertebra (L3) is the gold standard.^4^, ^5^ Aging, gender, sedentary lifestyle and malnutrition are major risk factors for sarcopenia. In head‐and‐neck squamous cell carcinomas (HNSCC), malnutrition is highly common, up to 46%–49% of patients at diagnosis^6^, ^7^ and sarcopenia emerges as an independent prognostic factor. Sarcopenia was associated with various negative clinical outcomes such as poor survival, increased chemotherapy dose‐limiting toxicity, treatment interruptions, hospital stay and post‐surgical complications after total laryngectomy.^8^, ^9^, ^10^, ^11^, ^12^, ^13^, ^14^, ^15^, ^16^, ^17^, ^18^, ^19^ However, there is a lack of study on the association between sarcopenia and survival in HNSCC.

The standard conservative treatment option for most locally advanced HNSCC relies on chemoradiotherapy, which is associated with significant side effects such as oral mucositis, xerostomia, dysgeusia, dysphagia, vomiting, cervical fibrosis and mandibular osteoradionecrosis.^20^, ^21^ All these side effects often require unscheduled treatment interruptions in order to recover and heal. These interruptions allow accelerated repopulation and increase radioresistance which can lead to worse outcomes.^22^

Little information exists regarding the correlation between sarcopenia and chemo‐induced toxicity^4^, ^9^, ^15^ and radiation‐induced toxicity.^10^, ^11^, ^15^ Therefore, the purpose of our current observational study designed according to the guidelines of STROBE was to assess the relationship between pre‐therapeutic sarcopenia and survival and its impact on treatment tolerance.

## 2 MATERIALS AND METHODS

### 2.1 Study design

The main endpoint of this observational study was to assess the relationship between pre‐therapeutic sarcopenia and survival (overall survival [OS] and disease‐free survival [DFS]) in patients with HNSCC treated with definitive chemoradiotherapy in the radiotherapy department of our hospital.

The secondary endpoint was to evaluate the impact of sarcopenia on treatment tolerance.

### 2.2 Population

Between 2009 and 2018, 332 consecutive patients with HNSCC treated by definitive radiotherapy combined with systemic therapy (cisplatin, carboplatin, 5‐fluoro‐uracil [5‐FU] or cetuximab) in the radiotherapy department of our hospital were examined for eligibility for the study. The recruitment period has been defined to achieve at least a 3‐year follow‐up.

The inclusion criteria were as follows: histologically confirmed diagnosis of squamous cell carcinoma, positron emission tomography–computed tomography (PET/CT) scans available at diagnosis, treatment by chemoradiotherapy with at least one concomitant cycle with curative intent. Prior neoadjuvant chemotherapy and/or surgery were allowed.

Exclusion criteria included metastatic disease, absence of PET/CT at diagnosis, no concomitant chemotherapy, palliative radiotherapy, current or previous malignancy with less than 3 years of complete remission (except non‐melanoma skin cancers and in‐situ cancers).

### 2.3 Treatment characteristics

Radiation therapy used three‐dimensional conformal radiotherapy (3D‐CRT), or volumetric arc therapy (VMAT; RapidarcR) delivered by Linac (VarianR) to a total dose of 66–72 Gy (median: 70 Gy) with classical fractionation of 1.8–2 Gy per fraction in 6–7 weeks as previously published^23^, ^24^, ^25^, ^26^ on high‐risk tumor and nodal volumes. The median prophylactic dose was 54.25 Gy. Concurrent chemotherapy (cisplatin, carboplatin, 5‐FU) was administered, or cetuximab if chemotherapy was contraindicated. Induction chemotherapy was offered to patients, who are selected for the organ preservation approach and at the discretion of the patient's medical oncologist. Induction chemotherapy was based on taxanes and platine and/or 5‐FU combination.^27^, ^28^

### 2.4 Clinical parameters

All the following variables were retrospectively collected from the medical file: age, gender, smoking status, alcohol use, weight, height, body mass index (BMI), performance status (PS) and albumin at baseline, primary tumor location, TN stage (according to the TNM and the 7th edition of the American Joint Committee on Cancer Staging Manual),^29^ human papilloma virus (HPV) status, treatment modalities, chemotherapy and radiation treatment interruptions and delays, types of nutritional support, treatment‐induced toxicity (according to the Common Terminology Criteria for Adverse Events [CTCAE, v4.03]), status at the latest news (alive or dead), date and cause of death.

### 2.5 CT image analysis

Muscle mass, subcutaneous fat and peri‐visceral fat were measured by the analysis of the L3 entire vertebral arc from the diagnostic PET/CT images, using a macro^30^, ^31^ developed for the National Institutes of Health software Image J (https://imagej.nih.gov/ij/download.html).^32^

**FIGURE 1:**
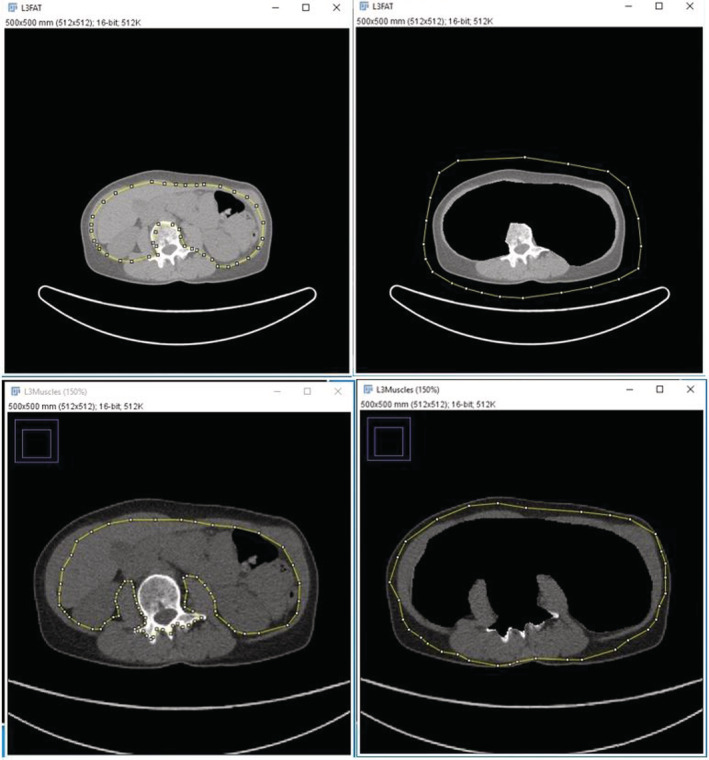
Examples of skeletal muscle contours. Pictures are example contours of segment manually muscles, subcutaneous adipose tissue and visceral adipose tissue at level L3 on computed tomography.

Figure 1 represents an example of skeletal muscle contours on a transversal CT slice at the level of L3 of CT images. The radiation oncologist who performed, twice for verification, these measurements was blinded to the treatment outcomes of all patients to minimize bias. The Hounsfield unit (HU) setting ranged from −29 to +150 HU for the muscle area, from −190 to −30 HU for the subcutaneous fat and the peri‐visceral fat.

The cross‐sectional muscle area (CSA) slide (cm^2^) was measured at the level of L3. The CSA (cm^2^) for each image was related to the body area to establish a skeletal muscle index (SMI, in cm^2^/m^2^). The SMI was calculated by dividing the skeletal muscle area by the squared height. Sarcopenia cut‐off values were set at SMI <43.3 cm^2^/m^2^ in men and <33.09 cm^2^/m^2^ in women (lowest gender specific quartile values of our population) according to a recent publication.^18^

### 2.6 Outcome measures

Outcome measures included OS, DFS and treatment tolerance (treatment‐induced acute and late toxicity, and treatments unscheduled interruption).

For pre‐ and per‐therapeutic nutritional status assessment, following definitions were used: malnutrition was defined by a weight loss >5% during treatment and/or a plasmatic albumin rate <35 g/L and/or a reduced BMI <18.5 kg/m^2^ and/or <21 kg/m^2^ in patients younger and older than 70 years, respectively.

### 2.7 Follow‐up

Follow‐up was calculated from the date of the end of treatment to the date of the last information. During treatment, all grades of acute toxicity were assessed weekly. After treatment, follow‐up was conducted every 3 months during the first year then every 6 months thereafter up to 60 months. Follow‐up included clinical examination at each time and imaging by magnetic resonance imaging or CT scan, depending on the initial tumor site. Late‐toxicity Grades 3 and 4 were carefully documented.

Time‐to‐event was defined from the last day of radiotherapy to the date of the event (death or recurrence). Patients who did not have an event or were alive at the time of the last follow‐up were censored in April 2020.

### 2.8 Data analysis

Descriptive statistics were used to summarize patient's characteristics using number of observations, percentages or mean (standard deviation) or median (interquartile range). Comparisons between groups were assessed by use of the *χ* ^2^ test or Fisher's exact test if the hypothesis of chi‐squared was not respected for qualitative variables and were assessed by student test or Wilcoxon–Mann–Whitney if the hypothesis of student test was not respected for continuous variables. The normality hypothesis was checked by Shapiro–Wilks test.

The median follow‐up was described using the reverse Kaplan–Meier method, OS and DFS curves calculated for patients with or without sarcopenia were plotted according to the Kaplan–Meier method and compared using the log‐rank test or Wilcoxon tests in case of crossing survival curves. Patients alive were censored at the date of the last news. The association of variables with the outcome (OS and DFS) was investigated using Cox proportional hazards regression analysis and expressed as hazard ratio (HR) with their 95% confidence interval (CI). Sarcopenia, age, gender, WHO PS, weight, BMI, albumin, smoking and alcohol history, HPV status, tumor stage and location, treatment modalities and radiotherapy technique were judged as clinically relevant and were included in the univariate analysis. Variables with *p* < 0.10 were added to the multivariate analysis as potential confounders.

All applied statistical tests were two‐sided, and a *p*‐value less than 0.05 were considered statistically significant.

Statistical analyses were performed using R Studio version 3.6.1.

## 3 RESULTS

### 3.1 Participants and treatments

Between 2009 and 2018, 216 patients with HNSCC treated by definitive radiotherapy combined with systemic treatment met the inclusion criteria and were included in the study for analysis.

The median follow‐up was 46.5 months (range: 3–129 months). Patient characteristics and missing data are presented in Table 1. Two hundred patients (94.7%) presented with stages III–IV cancer. The oropharynx was the main location reported (47.1%). HPV infection was seen in 64 patients.

**TABLE 1:** Baseline characteristics and differences of proportions and mean values between patients with and without sarcopenia

Covariate	Total (*n* = 212)	Non‐sarcopenic (*n* = 158)	Sarcopenic (*n* = 54)	*p*‐Value
Gender				
Female	36 (16.98%)	27 (17.09%)	9 (16.67%)	0.943
Male	176 (83.02%)	131 (82.91%)	45 (83.33%)	
Mean age at diagnosis	60.36 (±9.4) (37–83)	59.71 (±9.53) (37–83)	62.28 (±8.83) (41–81)	0.062
Alcohol history	91 (42.92%)	61 (38.61%)	30 (55.56%)	**0.03**
Smoking history	161 (75.94%)	113 (71.52%)	48 (88.89%)	**0.01**
Smoking cessation	111 (69.38%)	73 (65.18%)	38 (79.17%)	0.079
Histology				0.38
Epidermoid	192 (90.57%)	144 (91.14%)	48 (88.89%)	
UCNT	19 (8.96%)	14 (8.86%)	5 (9.26%)	
Adenocarcinoma	1 (0.47%)	0 (0%)	1 (1.85%)	
Localisation				
Oropharynx	100 (47.17%)	77 (48.73%)	23 (42.59%)	0.683
Hypopharynx	17 (8.02%)	11 (6.96%)	6 (11.11%)	
Larynx	63 (29.72%)	46 (29.11%)	17 (31.48%)	
Oral cavity	5 (2.36%)	3 (1.9%)	2 (3.7%)	
Nasopharynx	27 (12.74%)	21 (13.29%)	6 (11.11%)	
HPV status				**0.011**
Positive	64 (47.76%)	43 (41.75%)	21 (67.74%)	
ND	78	55	23	
EBV status nasopharynx tumor (*n* = 27)				1
Positive	15 (78.9%)	11 (78.5%)	4 (80%)	
ND	8	7	1	
T				0.653
Tis	1 (0.48%)	1 (0.64%)	0 (0%)	
T1	19 (9%)	16 (10.1%)	3 (5.6%)	
T2	40 (19.05%)	33 (21.02%)	7 (13.21%)	
T3	66 (31.43%)	47 (29.94%)	19 (35.85%)	
T4	84 (40%)	60 (38.22%)	24 (45.28%)	
Missing	2	1	1	
*N*				0.869
N0	56 (26.79%)	41 (26.28%)	15 (28.3%)	
N1	33 (15.79%)	25 (16.03%)	8 (15.09%)	
N2	105 (50.24%)	80 (51.28%)	25 (47.17%)	
N3	15 (7.18%)	10 (6.41%)	5 (9.43%)	
Missing	3	2	1	
Clinical stage^a^				0.433
II	11 (5.2%)	7 (4.4%)	4 (7.5%)	
III	51 (24.4%)	40 (25.64%)	11 (20.75%)	
IV	147 (70.33%)	109 (69.87%)	38 (71.7%)	
NC	3	2	1	
WHO‐PS at baseline				**0.024**
0	141 (66.51%)	113 (71.52%)	28 (51.85%)	
1	64 (30.19%)	40 (25.32%)	24 (44.44%)	
2	7 (3.3%)	5 (3.16%)	2 (3.7%)	
Neo‐adjuvant CT	191 (90.09%)	144 (91.14%)	47 (87.04%)	0.384
Surgery	42 (19.81%)	31 (19.62%)	11 (20.37%)	0.905
Type of surgery				0.699
T	6 (14.29%)	4 (12.9%)	2 (18.18%)	
N	23 (54.76%)	18 (58.06%)	5 (45.45%)	
T and N	13 (30.95%)	9 (29.03%)	4 (36.36%)	
RT technique				0.167
3D‐CRT	89 (41.98%)	62 (39.24%)	27 (50%)	
IMRT	123 (58.02%)	96 (60.76%)	27 (50%)	
Mean radiation total dose planned (Gy)	69.58 (±1.07) (66–72)	69.66 (±0.91) (66–70.2)	69.35 (±1.43) (66–72)	0.355
Mean radiation total dose received (Gy)	69.53 (±1.28) (60–72)	69.63 (±0.99) (66–72)	69.23 (±1.86) (60–72)	0.309
Mean RT treatment time (days)	54.37 (±7.75) (24–84)	54.68 (±7.73) (24–78)	53.48 (±7.81) (32–84)	0.709
Mean CT number of cycles received	6.5 (±1.66) (1–9)	6.6 (±1.58) (1–9)	6.22 (±1.86) (2–8)	0.275
Concurrent CT type				0.474
Cisplatine	79 (37.26%)	62 (39.24%)	17 (31.48%)	
Carboplatine	99 (46.7%)	71 (44.94%)	28 (51.85%)	
Cetuximab	27 (12.74%)	21 (13.29%)	6 (11.11%)	
Carboplatine‐cetuximab	6 (2.83%)	3 (1.9%)	3 (5.56%)	
Cisplatine‐5‐FU	1 (0.47%)	1 (0.63%)	0 (0%)	
Modifications of concurrent CT	10 (4.7%)	8 (5%)	2 (3.7%)	0.378

Abbreviations: 3D‐CRT, 3‐dimensional conformal radiotherapy; 5‐FU, 5‐fluoro‐uracil; CT, chemotherapy; EBV, Epstein–Barr virus; HPV, human papilloma virus; IMRT, intensity‐modulated radiotherapy; M, metastasis; N, node; ND, not done; RT, radiotherapy; T, tumor; UCNT, undifferentiated carcinoma nasopharyngeal type; WHO PS, World Health Organization performance score.

^a^ According to the staging proposed by the AJCC (8th edition).

Statistically significant values are shown in bold.

### 3.2 Clinical parameter comparison between patients with and without sarcopenia

#### 3.2.1 Sarcopenic versus non sarcopenic

Fifty‐four patients (25.47%) were defined as sarcopenic according to the sarcopenia cut‐off values.

As shown in Table 1, patients with sarcopenia were more likely to have a history of smoking (88.89% vs. 71.52%, *p* = 0.01), alcohol use (55.56% vs. 38.61%, *p* = 0.03) and positive HPV status (67.74% vs. 41.75%, *p* = 0.011). There were no significant differences in gender, age, tumor site, tumor histology, TN and clinical staging, PS, and treatment modalities between sarcopenic and non‐sarcopenic patients.

#### 3.2.2 Sarcopenia and nutritional status

The mean SMI in sarcopenic women was 29.56 cm^2^/m^2^ compared to 43.65 cm^2^/m^2^ in non‐sarcopenic women (*p* < 001). In male patients, the average SMI in patients with sarcopenia was 38.47 cm^2^/m^2^ compared to 53.76 cm^2^/m^2^ in patients without sarcopenia (*p* < 0.001).

Sarcopenic patients presented a lower mean BMI before treatment (21.92 vs. 25.65 cm/m^2^, *p* < 0.001) and experienced a significant weight loss >5% during treatment (9.26% vs. 1.9%, *p* = 0.027). Malnutrition was seen in 18 sarcopenic (33.96%) patients and 14 (9.15%) non‐sarcopenic patients (*p* < 0.001).

### 3.3 Outcome data

#### 3.3.1 Survival

**FIGURE 2:**
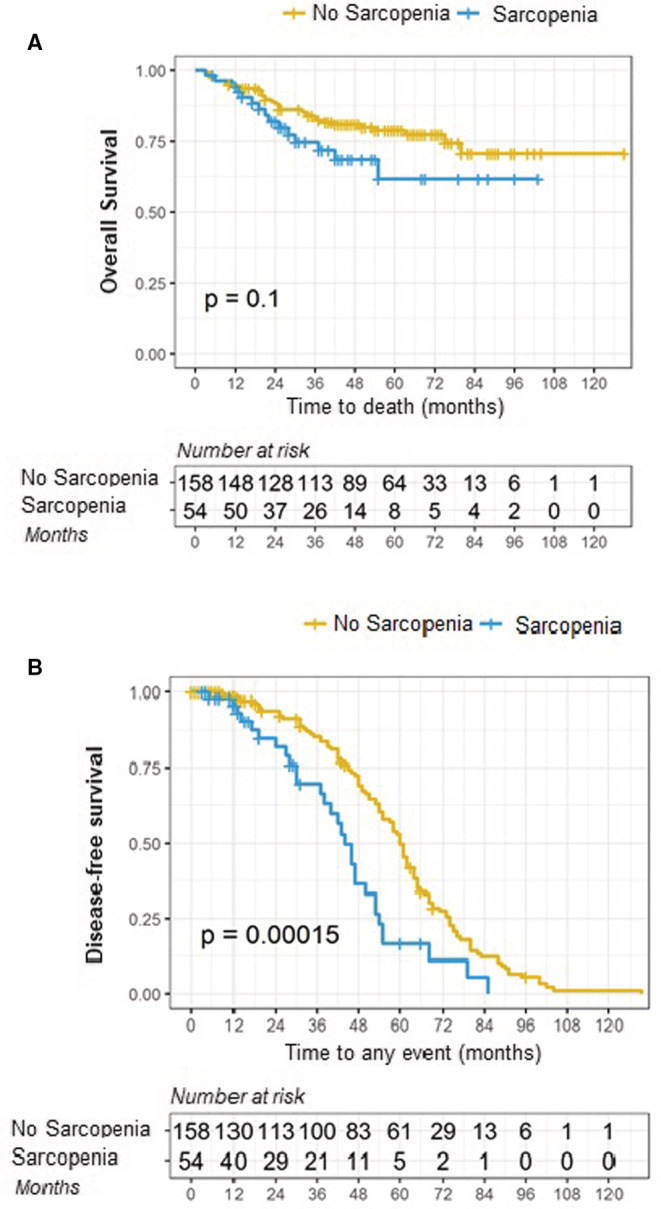
Kaplan–Meier analysis of (A) overall survival, *p* < 0.1 and (B) disease‐free survival, *p* < 0.00015.

Forty‐eight deaths (22.64%) were reported. Three‐year OS and DFS in sarcopenic patients were 75% and 70% versus 82% and 85% in non‐sarcopenic patients, respectively (*p* = 0.1 and *p* = 0.00015; see Figure 2A,B).

In univariate analysis, sarcopenia was a significant predictive factor for DFS (odds ratio = 2.191 95% CI 1.444–3.32, *p* = 0.0001) but not for OS (Table 2). After adjustment for confounders, Cox multivariate analysis failed to demonstrate a pejorative impact of sarcopenia on OS but confirmed its pejorative effect on DFS (Table 2). The HR of recurrence for sarcopenic patients was 2.174 times that of non‐sarcopenic patients (95% CI 1.431–3.302, *p* = 0.0001).

**TABLE 2:** Univariate and multivariate analysis of factors predicting overall survival and disease‐free survival

Variable	Overall survival (*n* = 212)	*p* Value	Disease‐free survival (*n* = 212)	*p* Value
	HR (95% CI)		HR (95% CI)	
Univariate analysis				
Gender (male vs. female)	1.619 [0.688; 3.81]	0.27	0.962 (0.63–1.471)	0.216
Age	1.035 (1.003–1.068)	**0.031**	1.015 (0.997–1.033)	0.097
BMI	0.961 (0.891–1.037)	0.305	0.99 (0.944–1.039)	0.687
WHO PS (0–1 vs. 2–3)	1.624 (0.393–6.71)	0.503	2.532 (1.11–5.778)	**0.027**
Smoking history (ever vs. never)	1.643 (0.769–3.512)	0.2	0.967 (0.663–1.391)	0.831
Alcohol history (ever vs. never)	2.018 (1.136–3.582)	**0.017**	0.886 (0.629–1.248)	0.489
HPV status (positive vs. negative)	0.313 (0.163–0.603)	**0.001**	0.842 (0.601–1.181)	0.32
Tumor stage (I–II vs. III–IVa)	3.168 (0.437–22.981)	0.254	1.138 (0.591–2.191)	0.7
Primary tumor site (larynx vs. other)	0.987 (0.529–1.842)	0.967	1.207 (0.832–1.752)	0.321
Primary tumor site (OP vs. other)	0.695 (0.389–1.24)	0.218	0.986 (0.708–1.373)	0.933
Sarcopenia (yes vs. no)	1.048 (0.964–1.14)	0.27	2.191 (1.444–3.324)	**0.0001**
Malnutrition according to				
BMI baseline (>18 vs. <18)	0.453 (0.162–1.266)	0.131	0.701 (0.286–1.717)	0.437
Albumin baseline (>35 vs. <35)	0.248 (0.129–0.478)	**0.0001**	1.025 (0.477–2.203)	0.949
Weight loss during RT (<−5% vs. >−5%)	1.764 (0.243–12.794)	0.575	0.945 (0.349–2.56)	0.911
Malnutrition pool	0.307 (0.166–0.567)	**0.0001**	0.927 (0.51–1.683)	0.802
Surgery (yes vs. no)	0.697 (0.312–1.555)	0.378	0.966 (0.65–1.435)	0.863
Neo‐adj. CT (yes vs. No)	2.447 (0.594–10.083)	0.216	0.867 (0.512–1.469)	0.596
Technique (IMRT vs. 3D‐CRT)	0.558 (0.316–0.986)	**0.044**	1.008 (0.714–1.422)	0.964
Multivariate analysis				
WHO PS (0–1 vs. 2–3)			2.439 (1.067–5.576)	**0.035**
HPV status (positive vs. negative)	0.338 (0.168–0.68)	**0.002**		
Primary tumor site (larynx vs. other)	0.496 (0.235–1.045)	0.065		
Sarcopenia (yes vs. no)			2.174 (1.431–3.302)	**0.0001**
Malnutrition according to				
Weight loss during RT (<−5% vs. >−5%)	5.984 (0.776–46.167)	0.086		
Malnutrition pool	0.298 (0.155–0.573)	**0.0001**		
Technique (3D vs. IMRT)	1.967 (0.263–0.984)	**0.045**		

Abbreviations: 3D‐CRT, 3‐dimensional conformal radiotherapy; BMI, body mass index; CI, confidence interval; CT, chemotherapy; HPV, human papilloma virus; HR, hazard ratio; IMRT, intensity‐modulated radiotherapy; RT, radiotherapy; WHO PS, World Health Organization performance score.

Statistically significant values are shown in bold.

#### 3.3.2 Treatment‐induced toxicity and completion

Ninety‐three patients (43.87%) experienced a chemotherapy interruption, and 32 patients required unscheduled radiation treatment interruption >1 week due to toxicity.

Transient radiation interruption and transient or definitive chemotherapy interruption frequency did not differ between sarcopenic and non‐sarcopenic patients (*p* = 0.444 and *p* = 0.677, respectively).

Grade 3 acute mucositis and radiation dermatitis were seen in 41 patients (19.34%) and 46 patients (21.7%). There was one Grade 4 mucositis and no toxic deaths.

No significant difference in Grade 3+ acute toxicities was found between sarcopenic and non‐sarcopenic patients. Sarcopenic patients were less likely to use analgesic (61.11% vs. 76.58%, *p* = 0.028) and did not use more nutritional support (*p* = 0.862) although significantly more undernourished (*p* = 0.001). Treatment completion and acute toxicities are reported in Table 3. Grade 3 mandibular osteoradionecrosis was seen in one patient. There were no Grade 4 late toxicity reported nor significant difference in Grade 3 late toxicity between sarcopenic and non‐sarcopenic patients.

**TABLE 3:** Treatment completion and acute toxicities

	Total	No sarcopenia (*n* = 158)	Sarcopenia (*n* = 54)	*p*‐Value
Treatment completion				
RT completion	169 (79.72%)	124 (78.48%)	45 (83.33%)	0.444
Transient interruption	43 (20.28%)	34 (21.52%)	9 (16.67%)	
Mean RT delay (days)	9.67 (±5.94) (2–29)	9.3 (±5.65) (2–29)	11 (±7.12) (6–28)	0.608
RT interruptions ≥7 days	32 (74%)	25 (73.5%)	7 (77.7%)	1
Toxicity related interruption	34 (70.83%)	26 (74.29%)	8 (61.54%)	**0.019**
Concomitant CT completion	119 (56.13%)	90 (56.96%)	29 (53.7%)	0.677
Concomitant CT interruption	93 (43.87%)	68 (43.04%)	25 (46.3%)	
Mean CT interruption (cycle)	1.36 (±3.15) (1;6)	6.64 (±3.41) (1;6)	7.67 (±3.97) (4;6)	0.608
CT interruption ≥2 cycles	41 (97.62%)	32 (96.97%)	9 (100%)	1
Toxicity related interruption	90 (96.77%)	65 (95.59%)	25 (100%)	0.561
Acute toxicity				
Nausea				
No toxicity or grade 1–2	211 (99.53%)	157 (99.37%)	54 (100%)	1
Grade 3	1 (0.47%)	1 (0.63%)	0 (0%)	
Vomiting				
No toxicity or grade 1–2	211 (99.53%)	157 (99.37%)	54 (100%)	1
Grade 3	1 (0.47%)	1 (0.63%)	0 (0%)	
Diarrhea				
No toxicity or grade 1–2	212 (100%)	158 (100%)	54 (100%)	–
Grade 3	0 (0%)	0 (0%)	0 (0%)	
Anemia				
No toxicity or grade 1–2	212 (100%)	158 (100%)	54 (100%)	–
Grade 3	0 (0%)	0 (0%)	0 (0%)	
Neutropenia				
No toxicity or grade 1–2	209 (98.58%)	155 (98.1%)	54 (100%)	0.572
Grade 3	3 (1.42%)	3 (1.9%)	0 (0%)	
Thrombopenia				
No toxicity or grade 1–2	208 (98.11%)	154 (97.47%)	54 (100%)	0.574
Grade 3	4 (1.89%)	4 (2.53%)	0 (0%)	
Renal failure				
No toxicity or grade 1–2	209 (98.58%)	157 (99.37%)	52 (96.3%)	0.16
Grade 3	3 (1.42%)	1 (0.63%)	2 (3.7%)	
Mucositis				
No toxicity or grade 1–2	171 (80.66%)	124 (78.48%)	47 (87.04%)	0.169
Grade 3–4	41 (19.34%)	34 (21.52%)	7 (12.96%)	
Xerostomia				
No toxicity or grade 1–2	208 (98.11%)	154 (97.47%)	54 (100%)	0.574
Grade 3	4 (1.89%)	4 (2.53%)	0 (0%)	
Dysgeusia				
No toxicity or grade 1–2	184 (86.79%)	137 (86.71%)	47 (87.04%)	0.951
Grade 3	28 (13.21%)	21 (13.29%)	7 (12.96%)	
Radiation dermatisis				
No toxicity or grade 1–2	166 (78.3%)	124 (78.48%)	42 (77.78%)	0.914
Grade 3	46 (21.7%)	34 (21.52%)	12 (22.22%)	
Use of analgesic				
Yes	154 (72.64%)	121 (76.58%)	33 (61.11%)	**0.028**
Use of nutritional support				
Yes	96 (45.28%)	71 (44.94%)	25 (46.3%)	0.862
Nasogastric	3 (3.12%)	0 (0%)	3 (12%)	**0.032**
Gastrostomia	79 (82.29%)	60 (84.51%)	19 (76%)	
Parenteral	14 (14.58%)	11 (15.49%)	3 (12%)	

Abbreviations: CT, chemotherapy; RT, radiotherapy.

Statistically significant values are shown in bold.

## 4 DISCUSSION

The present observational cohort of 216 patients treated with definitive chemoradiation for HNSCC confirmed that pre‐therapeutic sarcopenia is an independent negative prognostic factor for DFS. The standard treatment for HNSCC is radiotherapy, with or without systemic treatment. Significant toxicities are associated with HNSCC treatment (oral mucositis, dysgeusia, dysphagia, odynophagia, xerostomia, and nausea and vomiting). All of these toxicities can lead to inadequate nutrition, subsequent malnutrition and weight loss which in turn results in sarcopenia. Sarcopenia is a poorly understood condition represented by a progressive and generalized degenerative loss of SMM, quality and function. The underlying explanation of how sarcopenia contributes to poorer outcomes is probably multiple. Several mechanisms may be involved in the effect of sarcopenia on cancer outcomes, and one of them may be that skeletal muscle has recently been identified as an endocrine organ. Myokines are one of several hundred cytokines or peptides that are produced and released by skeletal muscle (muscle fibers). They have autocrine, paracrine and endocrine functions.^33^ More than 3000 identified myokines^34^ have been identified and some of them have antitumor activity.^34^, ^35^, ^36^ For example, the myokine interleukin 6, released by skeletal muscle, is a natural killer cell in tumor surveillance.^34^ Other mechanisms have been proposed: increased susceptibility to nosocomial infections, differential distribution of chemotherapy volume, and systemic inflammation associated with a higher rate of metastasis.^37^, ^38^ These findings may explain why sarcopenia may have a worse oncological prognostic.

In solid cancer, sarcopenia has been associated with various negative clinical outcomes. Table 4 summarizes studies comparing sarcopenic patients versus non‐sarcopenic patients in head and neck malignancies treated with chemoradiotherapy. Data on sarcopenia in patients with head‐and‐neck cancer focus mainly on OS. To our knowledge, only few studies examine the DFS rates.^11^, ^12^, ^18^ van Rijn‐Dekker et al. reported 48% 3‐year DFS in sarcopenic patients versus 69% in non‐sarcopenic patients (*p* < 0.001) in a large population of 750 HNSCC patients.^18^ Jung et al. found that sarcopenia was an independent variable that predicted for DFS after definitive treatments in patients with HNSCC (HR: 3.06, *p* < 0.05).^14^ In addition, Tamaki et al. reported a negative impact of sarcopenia on DFS in relation to HPV status in a retrospective cohort of 113 patients.^11^

**TABLE 4:** Summary of studies comparing sarcopenic patients versus non‐sarcopenic patients in head and neck malignancies treated with chemoradiotherapy (CRT)

Author/year	Number of patients, site	Survival Sarcopenic vs. non‐sarcopenic	Toxicity	Sarcopenia			
				Assessment of muscle mass	Spine level	Cutoff value (cm^2^/m^2^)	Prevalence (%)
van Rijin‐Dekker/2020	744, HP, L, OC, OP, others	Worse OS (HR: 0.72, *p* = 0.012) Worse DFS (HR: 0.67, *p* = 0.001)	Xerostomia 6 months after (OR 1.65, *p* = 0.027) dysphagia 6 and 12 months (OR 2.02, *p* = 0.012 and 2.51, *p* = 0.003)	SMI	C3	42.4 for male 30.6 for female	25.4
Shodo/2020	41, HP, L, OP, other	2‐year lower DSS rate (61% vs. 97%, *p* = 0.012)	NA	SMI	L3	39.7	26.8
Lee/2020	174, OS	Worse OS (HR:: 2.12, *p* = 0.01) Worse DFS (HR: 1.68, *p* = 0.047)	NA	SMI	C3	52.4 for male 36.2 for female	31.0
Findlay/2020	79, HP, L, OC, OP, others	Worse OS (HR 2.0, *p* < 0.0001)	NA	SMI	L3	43 for male, BMI <25 53 for male, BMI >25 41 for female	53.2
Choi/2020	79, HP, OC, OP, others	Worse OS (HR: 3.1, *p* = 0.016) Worse RFS (HR = 4.3, *p* < 0.001)	NA	Skeletal muscle area from the level of C3 to the level of the first rib 607 cm^3^ for male 450 cm^3^ for female			13.9
Ganju/2019	246, HP, L, OP, others	Worse OS (HR 1.83, *p* = 0.03) and PFS (HR 1.65, *p* = 0.03)	NA	SMI	C3	43 for male, BMI <25 53 for male, BMI >25 41 for female	58.1
Tamaki/2019	113, OP	No association with OS (HR: 1.943, *p* = 0.050)	NA	SMI		43 for underweight or normal weight male 41 for obese males 41 for female	28
Chargi/2019	85, OP, HP, L, OC, others	Worse median OS (12.07 vs. 13.60 months, *p* = 0.02)	NA	SMI	C3	43.2	48.2
Ra Jung/2019	258, OP, OC, L, HP	Worse OS (HR: 3.93, *p* < 0.001)	NA	SMI	L3	52.4 for men 38.5 for women	6.6
Cho/2018	221, OP, HP, L, OC, others	Worse OS and PFS	NA	SMI	L3	49 for male 31 for female	48.0
Wendrich/2017	112, HP, L, OP, others	NA	More CDLT (44.3% vs. 13.7%, *p* < 0.001)	SMI	C3	43.2	54.5
Grossberg/2016	190, HP, L, OC, OP, others	Worse OS (HR: 1.92, *p* = 0.007)	NA	SMI	L3	52.4 for male 38.5 for female	35.3

Abbreviations: BMI, body mass index; C3, third cervical vertebra; CDLT, chemotherapy dose‐limiting toxicity; CRT, chemoradiation therapy; DSS, disease‐specific survival; HP, hypopharynx; HR, hazard ratio; L, larynx; L3, third lumbar vertebra; OC, oral cavity; OP, oropharynx; OS, overall survival; PFS, progression‐free‐survival; RFS, recurrence‐free survival; SMI, skeletal muscle index.

In contrast to other cited studies,^8^, ^9^, ^10^, ^11^, ^12^, ^13^, ^14^, ^15^, ^16^, ^17^, ^18^, ^19^ our study failed to demonstrate the prognostic value of sarcopenia on OS, possibly because the third lumbar vertebral level, which was used to determine sarcopenia, was less accurate than the C3 vertebral level. Moreover, we believe that sarcopenia may be reversed by local tumor regression following treatment, which might explain why we found a significant difference in DFS but not in OS. Similarly, in a cohort of 190 HNSCC patients treated with chemoradiotherapy, Grossberg et al. showed that sarcopenia shortened OS (75% and 62% in sarcopenic and non‐sarcopenic patients, respectively).^8^ A recent meta‐analysis of published data which included 11 studies including the one mentioned above and 2483 patients, found that the sarcopenic patients had a significantly worse OS compared to the non‐sarcopenic patients (HR: 2.15, *p* = 0.04).^39^ This difference might be due to the SMM estimation method at L3 or C3 level and the different SMI cut‐off value. In fact, most studies had measured SMM on routine head and neck CT scan at a C3 level. Swartz et al. compared SMM at the L3 level and paravertebral muscle area at the third cervical vertebrae level using routine neck CT: CSA on the C3 level strongly predicted L3 muscles.^40^ In a recent study, SMI at C3 was strongly correlated with L3 level with thresholds of 14.0 cm^2^/m^2^ (men) and 11.1 cm^2^/m^2^ (women). Assessing SMM with routine neck CT scans might facilitate the screening of sarcopenia in practice use.^41^ Assessing SMM with routine neck CT scans might facilitate the screening of sarcopenia in practice use.

A valid cut‐off value for SMM for all oncological patients does not appear to be defined clearly or applicable for all patients with sarcopenia. Across studies, many different methods exist to establish a cut‐off value. The most common method of defining sarcopenia was to establish a cut‐off value intrinsic to the study: Below the lowest quartile for gender, below the study median population, below the fifth percentile for gender or below one standard derivation from the sex‐based mean. At least four studies defined sarcopenia as the lowest quartile of the CSA or SMI of their study population. However, in terms of generalizing findings, using an intrinsic cut‐off value is limited since the studies population might not reflect the larger sarcopenic population. Other studies used a gender‐specific cut‐off value extrinsic to the cohort, taken from other studies.

In our cohort, female patients in our cohort had significantly lower SMI scores than males; therefore, we used a gender‐specific cut‐off value. Following the largest cohort of van Rijn‐Dekker et al.^18^ and the most common method, we decided to use a cut‐off value corresponding to the lowest 25th percentile, which was set at 43.3 cm^2^/m^2^ in men and 33.09 cm^2^/m^2^ in women. Patients classified below these values were defined as sarcopenic. However, the two cohorts were not comparable, especially with a different mean SMI. Wendrich et al. determined an optimal cut‐off value for low SMM set at 43.2 cm/m^2^ to predict chemotherapy dose‐limiting toxicity.^9^ With this cut‐off, the majority of our cohort would have been classified as sarcopenic. Consequently, in order to establish both a uniform cut‐off and a methodology for assessing sarcopenia, a normative sex and age‐adjusted data curve from a healthy adult population would be helpful. Our study highlighted the importance of defining a standard cut‐off value for sarcopenia for HNSCC patients, especially in HPV‐positive oropharyngeal cancer patients who have few comorbidities.

Sarcopenic patients in our population were more likely to have a positive HPV status (*p* < 0.011). It is well known that HPV, commonly described to associate with younger patients without traditional risk factors, significantly improves OS and DFS.^42^ This may have contributed to a better outcome in the sarcopenic group.

In our study, no significant association was found between pre‐therapeutic sarcopenia and treatment tolerance. Acute grade 3+ toxicity was observed with similar frequency in both groups, and we did not observe more treatment unscheduled interruptions in sarcopenic patients. Nearly 13% of patients with sarcopenia and 16% of patients without sarcopenia required a treatment interruption >1 week. These rates were comparable to those seen in other studies.^15^, ^43^ Wendritch et al. reported that sarcopenia is an independent risk factor for chemotherapy dose‐limiting toxicity from platinum‐based concurrent chemoradiotherapy for locally advanced HNSCC (44% in sarcopenic patients vs. 14% in non‐sarcopenic patients).^9^ These results are largely consistent with those of Ganju et al. who showed that sarcopenia decreases compliance and increases toxicity in patients with HNSCC (45% of sarcopenic patients had dose‐limiting chemotherapy toxicities).^15^ In a cohort of 39 patients, Nishikawa et al. found that sarcopenia was not significantly associated with acute toxicities (mucositis, radiodermatitis and aspiration pneumonia) and late toxicities (hypothyroidism, dysgeusia and xerostomia).^10^ van‐Rijn Dekker et al. had investigated the correlation between sarcopenia and late radio‐induced toxicities. Sarcopenia was significantly correlated with Grade 2 physician‐rated xerostomia 6 months after treatment, and Grade 2 physician‐rated dysphagia at 6 and 12 months after treatment.^18^

Our study had some limitations. First, our conclusions are drawn from a single center retrospective study and may therefore be biased. Our study targeted locally advanced head and neck cancer with several different tumor types receiving chemoradiotherapy; thus, the conclusions are based on this heterogeneity characteristic of the patients. The use of various treatment regimens such as surgery and/or induction chemotherapy has the potential to bias the results of the present study. Most oncologists (90%) used induction chemotherapy with the hope of reaching a better local control and less metastatic extent, or in an organ preservation approach. There could also be a selection bias for patients treated with 3D‐CRT rather than IMRT because of limited access to IMRT. The study size was limited by the number of pre‐therapeutic PET/CT available.

Sarcopenia might be a major modifiable risk factor prior to treatment. Early detection of sarcopenia and early treatment such as intensive nutritional intervention,^44^ daily aerobic physical activity and resistance exercise could improve outcomes.^45^

## 5 CONCLUSION

Sarcopenia is a new independent prognostic factor in HNSCC patients and was, associated with poor DFS after definitive treatment, in our study. Conversely, sarcopenia was not a significant factor for either OS or poorer tolerance of chemotherapy and radiation treatments. Regardless, SMM is easily measured on the PET/CT images; physicians should assess sarcopenia in their clinical practice. Sarcopenia gives important and useful information about the patient's prognosis and can be used for patient counseling and treatment decision‐making.

## AUTHOR CONTRIBUTIONS

**Rita Bentahila:** Collection and compilation of data, formal analysis, writing – original draft, writing – review and editing. **Catherine Durdux:** Conception and design of the study, methodology, formal analysis, writing – original draft, writing – review and editing, supervision. **Philippe Giraud:** Conception and design of the study, methodology, formal analysis, writing – original draft, writing – review and editing, supervision. **Pierre Decazes:** Conceptualization, methodology, supervision. **Sarah Kreps:** writing – review and editing. **Emmanuelle Fabiano:** Writing – review and editing. **Paula Nay:** methodology. **Augustin Chatain:** methodology.

## CONFLICT OF INTEREST

The authors declare no conflict of interest.

## INSTITUTIONAL REVIEW BOARD STATEMENT

The study was conducted in accordance with the Declaration of Helsinki, and approved by the Ethics Committee of AP‐HP Centre (Réf. 2021‐12‐01) in Paris, on 7/1/2022.

## INFORMED CONSENT STATEMENT

Informed consent was obtained from all subjects involved in the study.

## Data Availability

Research data are stored in an institutional repository and will be shared upon request to the corresponding author.
